# Induction of TGF-β1 Synthesis by Macrophages in Response to Apoptotic Cells Requires Activation of the Scavenger Receptor CD36

**DOI:** 10.1371/journal.pone.0072772

**Published:** 2013-08-02

**Authors:** Weipeng Xiong, S. Courtney Frasch, Stacey M. Thomas, Donna L. Bratton, Peter M. Henson

**Affiliations:** 1 Department of Pediatrics, National Jewish Health, Denver, Colorado, United States of America; 2 Department of Immunology, University of Colorado Denver, Aurora, Colorado, United States of America; Massachusetts General Hospital and Harvard Medical School, United States of America

## Abstract

**Background/Objective:**

Phosphatidylserine (PS) exposed on apoptotic cells has been shown to stimulate production of transforming growth factor-β (TGF-β) and promote anti-inflammatory responses. However, the PS receptor(s) responsible for this induction has not been clearly determined.

**Methodology/Principal Findings:**

In the present study, using RAWTβRII cells in which a truncated dominant negative TGF-β receptor II was stably transfected in order to avoid auto-feedback induction of TGF-β, we show that TGF-β1 synthesis is initiated *via* activation of the scavenger receptor, CD36. The response requires exposure of PS on the apoptotic cell surface and was absent in macrophages lacking CD36. Direct activation of CD36 with an anti-CD36 antibody initiated TGF-β1 production, and signaling pathways involving both Lyn kinase and ERK1/2 were shown to participate in CD36-driven TGF-β1 expression.

**Conclusion/Significance:**

Since CD36 has been previously implicated in activation of secreted latent TGF-β, the present study indicates its role in the multiple steps to generation of this important biological mediator.

## Introduction

Clearance of apoptotic cells (efferocytosis [1–5]) is critical for tissue homeostasis and resolution of inflammation. Furthermore, recognition of apoptotic cells by potential phagocytes also leads to the generation of anti-inflammatory mediators [6–9], and the establishment of a generally anti-inflammatory and pro-resolution local environment. It has been suggested that TGF-β1 is a major mediator of this response, and that a number of secondary anti-inflammatory effects result from the autocrine/paracrine actions of the active TGF-β1 produced [7,8].

The TGF-β family comprises more than 60 structurally related growth and differentiation factors that play important roles in regulation of numerous physiological processes, including cell proliferation, differentiation, apoptosis, early embryonic development, and extracellular matrix protein synthesis [10–13]. TGF-β exerts its effects through a heteromeric receptor complex consisting of type I and II transmembrane serine/threonine kinase receptors [14]. In mammals, TGF-β exists in at least three isoforms, which are structurally identical and have similar, though not identical, bioactivities. Our previous studies showed TGF-β may be generated as a result of apoptotic cell interaction with inflammatory cells, such as macrophages, resulting in accelerated resolution of ongoing inflammation [7,15].

Recognition of apoptotic cells involves surface changes on the dying cells, in particular exposure of phosphatidylserine (PS). This anionic phospholipid is normally restricted to the inner membrane leaflet, but exposed on the outer leaflet as a consequence of loss of membrane phospholipid asymmetry during apoptosis [16,17]. There is considerable evidence to support a major role for recognition of PS in the production of TGF-β and the anti-inflammatory effects of apoptotic cells [7,8,18–21]. Thus, in our previous studies, we provided evidence that interaction of macrophages with apoptotic cell PS resulted in production of active TGF-β both in vitro and vivo [7,8,15,18]. On the other hand, although a wide spectrum of candidate receptors recognizing PS have been implicated in the uptake of apoptotic cells, less attention has been given to the modes of PS recognition that are involved in the anti-inflammatory effects and the induction of TGF-β synthesis. Thus, while uptake of apoptotic cells has been shown to involve receptors such as T-cell immunoglobulin and mucin domain-containing protein 4 (TIM4) [22,23], brain angiogenesis inhibitor 1 (BAI1) [24], stabilin-2 [25] or PS-recognizing bridge molecule-receptor combinations (e.g. growth arrest-specific 6 (GAS6) and Mer tyrosine kinase [26] or milk fat globule-EGF factor 8 protein (MFG-E8) and α_v_ integrins [27–29]), their possible role in inflammosuppression is not clear.

Accordingly, it was important to determine which PS receptor(s) contributes to apoptotic cell-induced TGF-β synthesis and release. CD36 is a member of the class B scavenger receptor family that is expressed on a variety of cell types and binds a diverse array of ligands [30]. It has also been identified as a PS receptor that can participate in apoptotic cell recognition and clearance [31–34]. Importantly, through its binding of thrombospondin, it has also been shown to participate in activation of secreted latent TGF-β [35,36]. Since PS recognition has also been shown to induce the synthesis of TGF-β, we have here explored the ability of CD36 to act as a key PS-recognizing receptor for mediation of synthesis and secretion of this mediator, i.e., as a candidate receptor for suppression of inflammation. Since TGF-β is not only active in inflammosuppression but also in fibroproliferative processes, the study additionally raises possible roles for this receptor in tissue remodeling and fibrosis [37,38].

The experiments herein used whole apoptotic cells as stimuli and show that PS-mediated interaction of apoptotic cells with CD36 induces TGF-β1 synthesis and release. In keeping with this role for CD36 the activating mouse IgA monoclonal antibody (JC63.1), known to selectively trigger CD36-driven internalization and signaling [39–41] was also shown to initiate TGF-β production. This latter system allows definition of early downstream signaling pathways involved in initiation of TGF-β1 synthesis that avoid the complexity of responses from engagement of additional apoptotic cell stimuli.

## Materials and Methods

### Antibodies and reagents

TGF-β1 was from R&D Systems (Minneapolis, MN). Phorbol myristate acetate (PMA), lipopolysaccharide (LPS), actinomycin D, cycloheximide, SB203580, U0126, U0124, JNK inhibitor, wortmannin and LY294002 were from Sigma Chemical Co. (St. Louis, MO). Protease Inhibitor Cocktail Set I, rapamycin and PP2 were obtained from Calbiochem-Novabiochem (Gibbstown, NJ). RNAiMAX, CD36 stealth RNAi™ siRNA, negative control LO GC siRNA, TRIzol reagent and gene specific relative RT-PCR kit were from Invitrogen (Grand Island, NY). Annexin V apoptosis detection kit FITC was from eBioscience (San Diego, CA). Purified annexin V used for blocking assay was purchased from BD Biosciences (San Jose, CA). Anti-CD36 (MF3) used for Western blotting assay, and the activating anti-CD36 mIgA (JC63.1) were purchased from ABCAM (Cambridge, MA). Mouse monoclone IgA was purchased from BD Biosciences (San Jose, CA). Extracellular signal-regulated protein kinases 1 and 2 (ERK1/2) antibody was purchased from Millipore (Billerica, MA). Phospho-ERK1/2 (Thr202) antibody was from Santa Cruz Biotech (Santa Cruz, CA). Anti-p38 mitogen-activated protein kinase (MAPK), phospho-p38 antibody (Thr180/Tyr182), phospho-c-Jun N-terminal kinase 1/2 (JNK1/2) antibody (Thr183/Tyr185), phospho-Akt (Thr308), phospho-Lyn (Tyr507), anti-Lyn (C13F9) antibodies were from Cell Signaling (Danvers, MA). Quantitative PCR SYBR® Green Master Mix was from Applied Biosystems (Foster City, CA).

### Cell culture and transfection

RAW 264.7 macrophage cell lines (ATCC, Manassas, VA) were stably transfected with truncated TGF-β receptor II (RAWTβRII) or empty vector (RAWV) [15]. Briefly, pcDNA3.1 plasmids (Invitrogen, Grand Island, NY) with or without MYC-tagged truncated TGF-β receptor II sequence were transfected into RAW 264.7 cells using Lipofectamine Plus reagent (Invitrogen, Grand Island, NY), according to the manufacturer’s instructions. Seventy-two hours after transfection, the cells were incubated in the fresh medium containing 500 µg/ml G418 for 4 weeks. Cell colonies resistant to G418 (Thermo, Fisher Scientific, Houston TX) were isolated and screened by limited dilution.

RAWTβRII or RAWV cells were plated at 5.0 X 10^5^ cells/ml and incubated overnight in Dulbecco’s modified Eagle’s medium (ATCC, Manassas, VA) supplemented with 10% FBS, L-glutamine (2 mM), penicillin (100 U/ml), streptomycin (100 mg/ml), and G418 (500 µg/ml). Cell lines were incubated under a humidified 5% CO_2_ atmosphere at 37 °C. RAWTβRII cells were transiently transfected with 50 nM targeting siRNA or control siRNA, premixed with RNAiMAX according to the manufacturer’s instructions (Invitrogen, Grand Island, NY). The cells were then incubated in fresh media for 24 h and subjected for further experimentation.

### Generation of apoptotic cells

Human Jurkat T cell line was obtained from ATCC (Manassas, VA) and grown in RPMI 1640 (Mediatech Inc., Manassas, VA) supplemented with 10% FBS, 2mM glutamine and antibiotics (100 units/ml penicillin, 100 µg/mls streptomycin) at 37° C in a humidified incubator with 5% CO_2_. Apoptotic cells were prepared as previously described [7]. Briefly, Jurkat T cells were exposed to UV irradiation at 254 nm for 10 min and cultured before performing experiments. Jurkat T cells were ~80% apoptosis by evaluation of nuclear morphology using light microscopy [42].

PS externalization was determined by a flow cytometry based annexin V staining [43]. Apoptotic cells (1×10^6^ cells) in 100 µl of 4-(2-hydroxyethyl)-1-piperazineethanesulfonic acid (HEPES) buffer (137mM NaCl, 2.7mM KCl, 2mM MgCl_2_, 5mM glucose, 10mM HEPES (pH 7.4)) supplemented with 2.5 mM CaCl_2_, were incubated with annexin V-Alexa 488 (1:50), and 5 µg/ml propidium iodide for 15 min at room temperature, diluted with 400 µl of ice-cold HEPES buffer supplemented with 2.5 mM CaCl_2_, and analyzed by flow cytometry.

### Quantitative PCR

Total RNA was isolated from cultured cells using TRIzol reagent (Invitrogen, Grand Island, NY). The concentration and purity of RNA were evaluated by spectrophotometry at 260 and 280 nm. Reverse transcription (RT) was carried out for 60 min at 37° C with 1 µg total RNA using MMLV RT (Invitrogen, Grand Island, NY). TGF-β1 mRNA level was determined using real-time PCR. The primer sequences were used as follows: mouse TGF-β1 (Forward: 5’-TGGAGCAACATGTGGAACTC-3’ and Reverse: 5’-TGCCGTACAACTCCAGTGAC-3’). Primer sequences of phagocytic receptors and bridge molecules were used as follows: mouse CD36 (forward: 5’-GAGGCATTCTCATGCCGTC-3’ and reverse: 5’-ACGTCATCTGGGTTTTGCAC-3’), Tim4 (forward: 5’-CACCAATCGAGGTGACAG-3’ and reverse: 5’-GACTGTTGTTGGAAGCAGC-3’), MFG-E8 (forward: 5′-ATCTACTGCCTCTGCCCTGA-3′ and reverse: 5′-ACACATACGAGGCGGAAATC-3′); CD91 (5′-ATCACCCTTCCCGGCAGCCCA-3′ and reverse 5′-ACCCAGAGCCATCGGCTTTGT-3′), C1qA (forward, 5′-ATGGAGACCTCTCAGGGATG-3′ and reverse 5′-ATACCAGTCCGGATGCCAGC-3′), C1qB (forward: 5’-ATAAAGGGGGAGAAAGGGCT-3’ and reverse: 5’-CGTTGCGTGGCTCATAGTT-3’), C1qC (forward: 5′-CGATACAAACAGAAGCACCAG-3’ and reverse: 5′-CTGGCAAGGTTGAGGTTCAG-3’), and mouse specific GAPDH (Forward: 5’-AACGACCCCTTCATTGAC-3’) and Reverse: 5’-TCCACGACATACTCAGCAC -3’). The cDNA was amplified by SYBR^®^ Green Master Mix (Applied Biosystems, Foster City, CA) to a final volume of 20 µl. The cDNA was denatured for 5 min at 94° C, followed by 40 cycles of temperature (95° C for 30 s, 52° C for 30 s, and 72° C for 45 s). The primers for GAPDH and TGF-β1 were used as described above. The Ct values of TGF-β1 were normalized to that of GAPDH. All samples were run in triplicate.

### Western Blot

The cultured cells were lysed in lysis buffer (20 mM of pH7.4 HEPES, 150 mM NaCl, 1 mM DTT, 0.5% Triton X-100 and 1 × Protease Inhibitor Cocktail Set I). Protein concentrations were determined by Bradford protein assay reagent (Bio-Rad, Hercules, CA). Equal amounts of proteins were separated by 10% SDS-PAGE and blotted to nitrocellulose membranes. After blocking, the membranes were incubated with primary antibodies at 4° C overnight. The membranes were washed and then incubated with horseradish peroxidase-conjugated secondary antibodies for 1 h at room temperature and proteins were visualized by enhanced chemiluminescence (Amersham Pharmacia Biotech, Piscataway, NJ) according to the manufacturer’s instructions.

### ELISA

Cultured RAWTβRII or RAWV cells were serum-starved in X-VIVO 10 (Lonza Inc., Allendale, NJ) for 1 h prior to stimulation. Secreted TGF-β1 in cell-free culture supernatant was measured by ELISA according to the instructions of the manufacturer (ELISA Tech, Denver, CO).

### Statistical analysis

Data are presented as means ± SD from five or more separate experiments. P-value calculations were conducted using a two-tailed *t* test for two group comparison or ANOVA (with Bonferroni posttest analysis for multiple comparisons). All data were analyzed and presented by using GraphPad Prism software (version 5.0; GraphPad Software Inc., La Jolla, CA) for the Macintosh.

## Results

### PS dependency of apoptotic cell stimulated TGF-β1 synthesis and secretion

**Figure 1:**
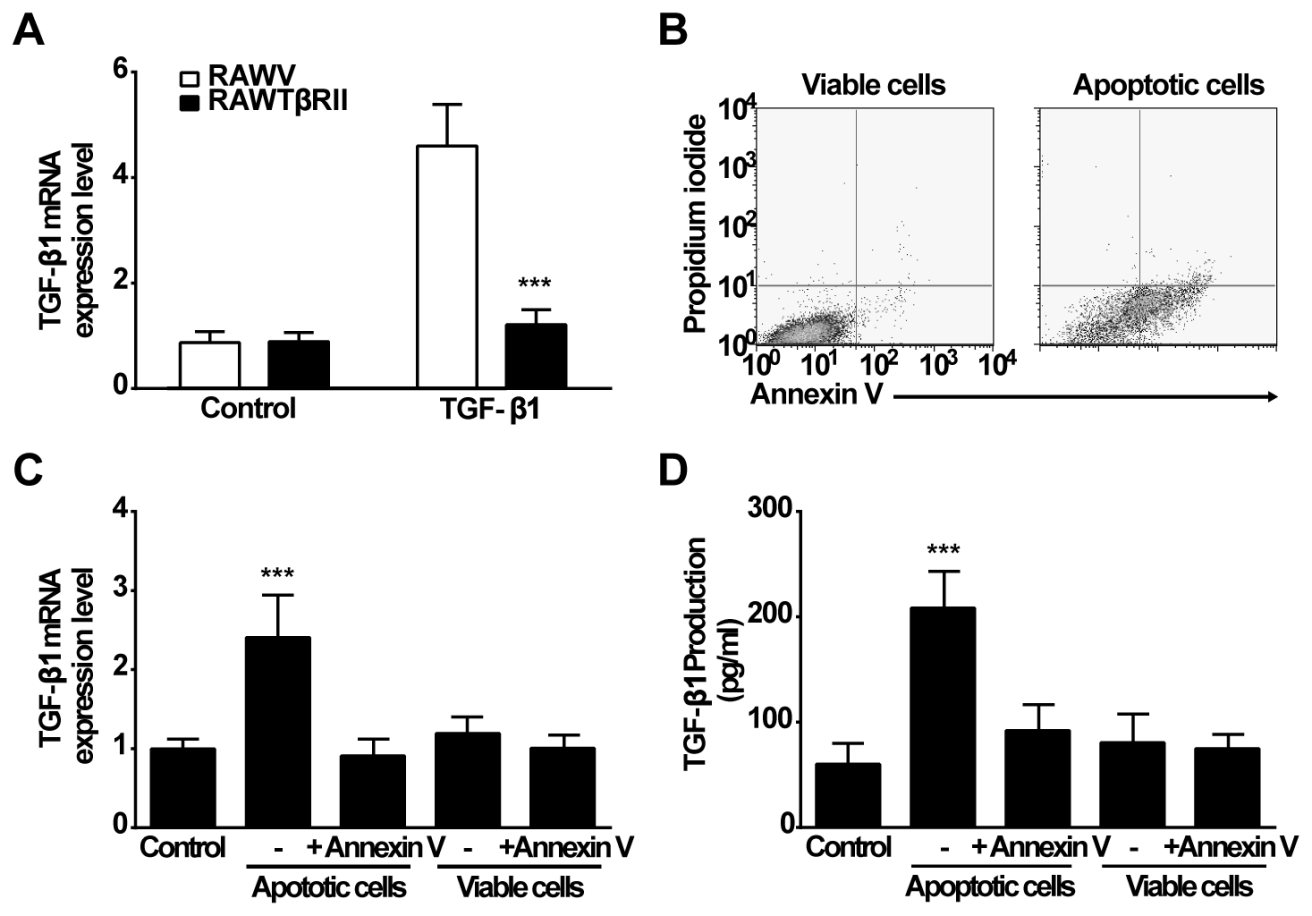
Apoptotic cells induce TGF-β1 production in RAWTβRII macrophages. A, RAW 264.7 macrophages were stably transfected with truncated TGF-β receptor II (RAWTβRII) or empty vector (RAWV). After 48 h, the cells were incubated with TGF-β1 (25 ng/ml) for 6 h. TGF-β1 mRNA expression which was normalized to GAPDH, was measured by real-time PCR and expressed as fold enhancement. B, Human Jurkat T cells were stimulated with UV-irradiation to induce apoptosis. Surface PS exposure was assessed by annexin V staining and cell permeability with propidium iodide as analyzed by flow cytometry (shown as a representative dot plot from five independent experiments). C, Apoptotic or viable Jurkat cells were pretreated with annexin V for 45 min, and incubated with RAWTβRII macrophages for 6 h to measure TGF-β1 mRNA expression. D, Total TGF-β1 in the conditioned medium was analyzed by ELISA after 18 h of co-culture. Values represent means ± SD of five separate experiments. ***, *P*<0.001.

To avoid auto-stimulation by TGF-β1 itself, a stable cell line unresponsive to TGF-β was created by transfecting truncated TGF-β receptor II constructs (or empty vector as control) into RAW 264.7 macrophages [15]. Blockade of TGF-β signaling was verified using PCR to measure TGF-β1 mRNA expression (Figure 1A). To confirm that PS recognition was required for production of TGF-β1, RAWTβRII cells were co-cultured with apoptotic Jurkat cells presenting surface exposed PS (Figure 1B) in the absence or presence of the PS-binding protein, annexin V. Incubation of RAWTβRII cells with apoptotic Jurkat cells resulted in enhanced TGF-β1 mRNA and secreted protein (Figure 1C and D), both of which were prevented by annexin V.

### TGF-β1 generation induced by apoptotic cells required CD36

**Figure 2:**
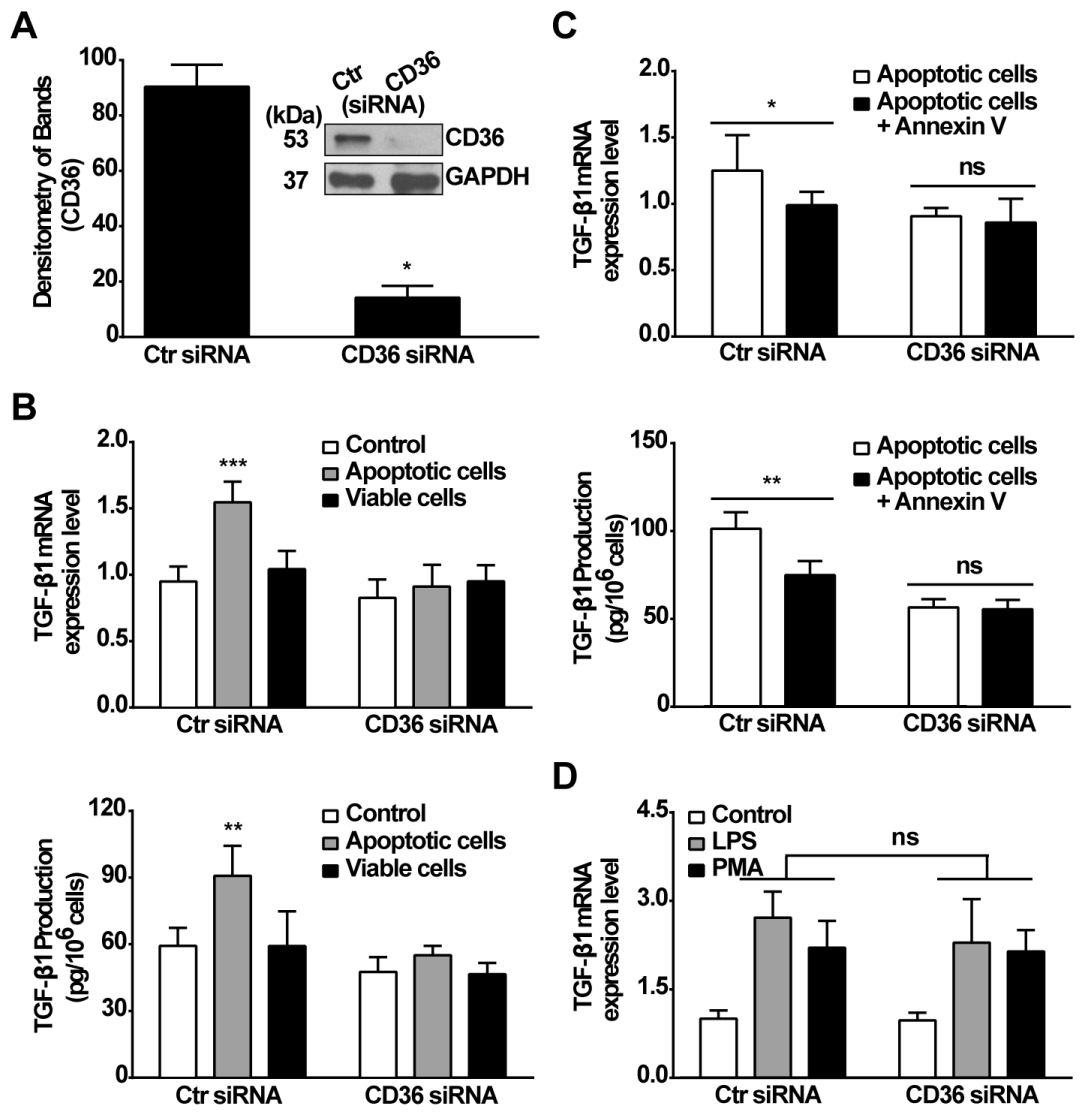
Receptor CD36 is required for apoptotic cells induced TGF-β1 mRNA and protein expression. A, CD36 protein expression in RAWTβRII cells transfected with CD36-target siRNA or control siRNA (Ctr siRNA) for 24 h was analyzed by Western blotting with anti-CD36 (MF3) antibody, and band density was normalized to GAPDH. B, RAWTβRII cells transfected with CD36-target siRNA or control vehicle (Ctr siRNA) for 24 h were stimulated with apoptotic or viable Jurkat cells. C, Apoptotic Jurkat cells were pretreated with annexin V for 45 min and incubated with RAWTβRII transfected with CD36-target siRNA or control siRNA (Ctr siRNA). TGF-β1 mRNA expression or secreted TGF-β1 protein was analyzed as in Figure 1. D, RAWTβRII cells transfected with CD36-target siRNA or control siRNA (Ctr siRNA) were treated with either LPS (25 ng/ml) or PMA (50 nM). The expression of TGF-β1 mRNA was analyzed as above. Values represent means ± SD of five separate experiments. ns, no significant; *, *P*<0.05; **, *P*<0.01; ***, *P*<0.001.

The role of CD36 in mediating apoptotic cell stimulated TGF-β1 generation was explored using knockdown with CD36-specific siRNA. RAWTβRII cells were transfected with CD36-specific siRNA or negative control siRNA for 24 h. The negative-control siRNA did not alter CD36 protein levels in RAWTβRII cells. However, CD36 protein levels after 24 h were markedly decreased (> 85%) in cells transfected with 50 nM CD36-specific siRNA (Figure 2A). CD36 levels returned to baseline levels after 48h (data not shown). TGF-β1 mRNA expression and secreted TGF-β1 protein were both blocked in the cells with suppressed CD36 (Figure 2B). To further confirm the role of exposed PS in recognition of apoptotic cells by CD36 mediated TGF-β1 production, annexin V was used to block PS recognition by the siRNA treated RAWTβRII cells. As shown in Figure 2C, annexin V treatment blocked the apoptotic cells induced TGF-β1 mRNA and protein production in control siRNA transfected RAW cells. However, TGF-β1 production was not further altered by annexin V treatment in CD36 knock-down RAWTβRII cells. Importantly, the CD36 defective cells were unchanged in their ability to synthesize TGF-β1 mRNA in response to the unrelated stimuli, LPS or PMA (Figure 2D). Furthermore, knock down of CD36 by RNA interference did not show any effects of off-target gene modulation on other phagocytic receptors and bridge molecules, i.e. TIM4, MFG-E8, CD91, C1q (Figure S1). These data strongly suggest recognition of apoptotic cells induces TGF-β1 production by macrophages through CD36.

### Stimulation with activating anti-CD36 mAb (JC63.1) induces TGF-β1 synthesis and secretion

**Figure 3:**
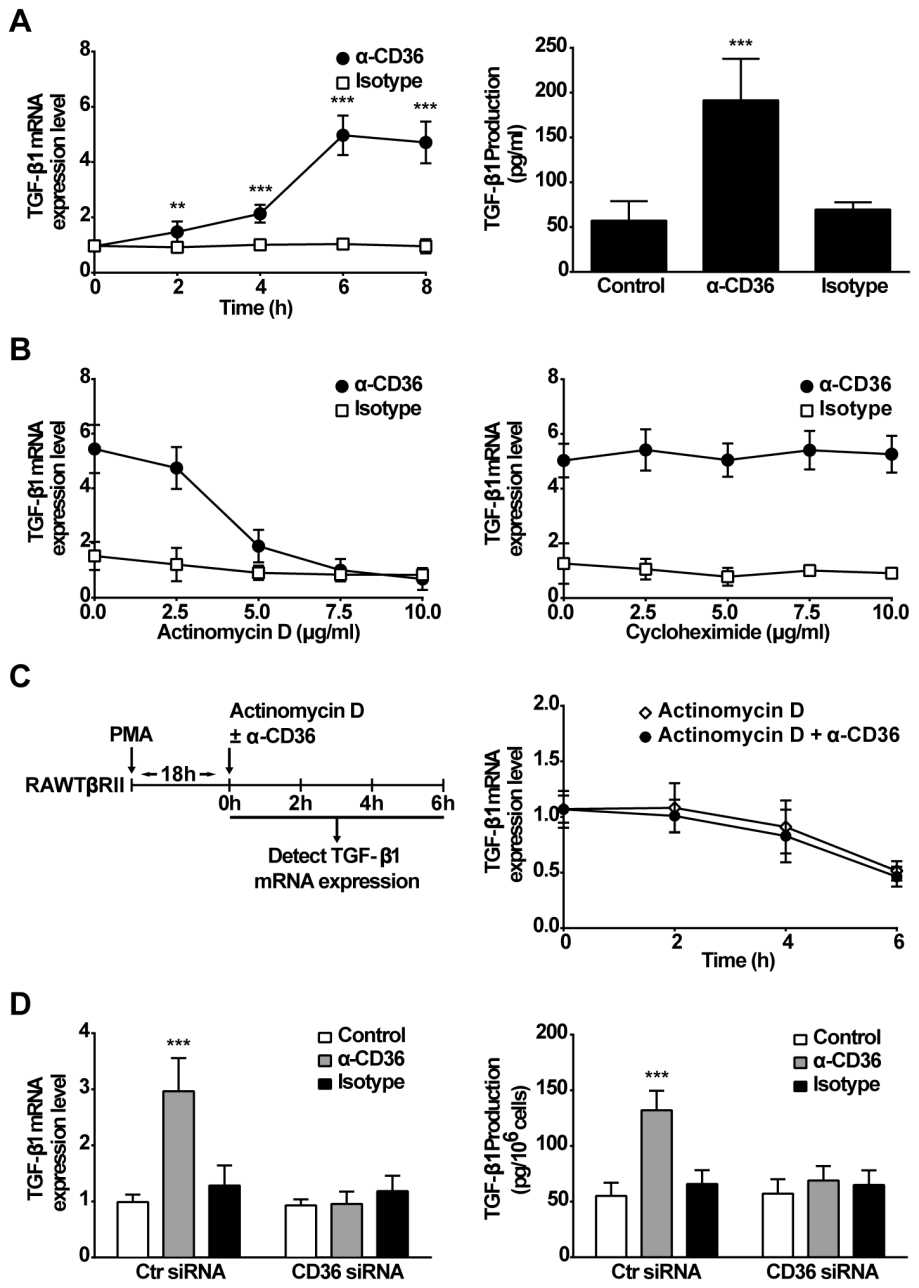
Stimulation with activating anti-CD36 mIgA induces TGF-β1 synthesis. A, RAWTβRII cells were cultured with activating anti-CD36 (JC63.1) mIgA or isotype control (2 µg/ml) for the times indicated. TGF-β1 mRNA expression or secreted TGF-β1 protein was analyzed as in Figure 1. B, RAWTβRII cells were pre-treated with the indicated concentrations of actinomycin D or cycloheximide for 1 h, before stimulation for 6 h and TGF-β1 mRNA expression was analyzed as above. C, RAWTβRII cells were cultured in the presence of PMA (50 nM) for 18 h to increase the steady state TGF-β1 mRNA level, and then the cells were incubated with actinomycin D (10 µg/ml) in the presence or absence of anti-CD36 mIgA (2 µg/ml) for the times indicated. D, RAWTβRII cells transfected with CD36-target siRNA or control siRNA (Ctr siRNA) for 24 h were incubated with anti-CD36 mIgA (2 µg/ml) or isotype control (2 µg/ml) for 6 h or 18 h to analyze TGF-β1 mRNA expression or secreted total TGF-β1 protein respectively, as in Figure 1. Values represent means ± SD of five separate experiments. **, *P*<0.01; ***, *P*<0.001.

Previous studies suggested CD36 is a multifunction receptor with potential anti-inflammatory properties [33,34,44,45]. To avoid the complexity of assay systems that may be confounded by the presence of both apoptotic as well as responder cells, RAWTβRII cells were incubated with activating anti-mouse CD36 mIgA (JC63.1) alone and TGF-β response was determined. As shown in Figure 3A, production of both mRNA and secreted TGF-β1 were increased, and showed a dose response in the presence of anti-CD36 mIgA. The isotype control had no effect. In addition, TGF-β1 mRNA expression was significantly inhibited by actinomycin D; however, not by the protein synthesis inhibitor, cycloheximide suggesting that new protein synthesis was not required for the induction of TGF-β1 transcription (Figure 3B). To rule out the possibility that the increase in TGF-β1 mRNA was caused by enhancement of TGF-β1 mRNA stability, RAWTβRII cells were first treated with PMA (50 nM) for 18 h to increase the steady state TGF-β1 mRNA level, and then washed and treated with actinomycin D (10 µg/ml) in the absence or presence of anti-CD36 mIgA. The remaining TGF-β1 mRNA level after actinomycin D treatment was measured using real-time PCR. As shown in Figure 3C, anti-CD36 did not affect TGF-β1 mRNA stability. These findings suggest that the upregulation is at the level of transcription. To further address that the induced TGF-β1 production is selectively dependent on CD36, RAWTβRII cells transfected with CD36 specific or control siRNA were tested. As expected, production of TGF-β1 mRNA and protein in response to the anti-CD36 stimulation was blocked in RAW cells transfected with CD36 specific siRNA (Figure 3D). These data strongly support our supposition that activation of CD36 contributes to up-regulate TGF-β1 production.

### Requirement for Lyn kinase and ERK1/2 MAPK in CD36 stimulated TGF-β1 production

**Figure 4:**
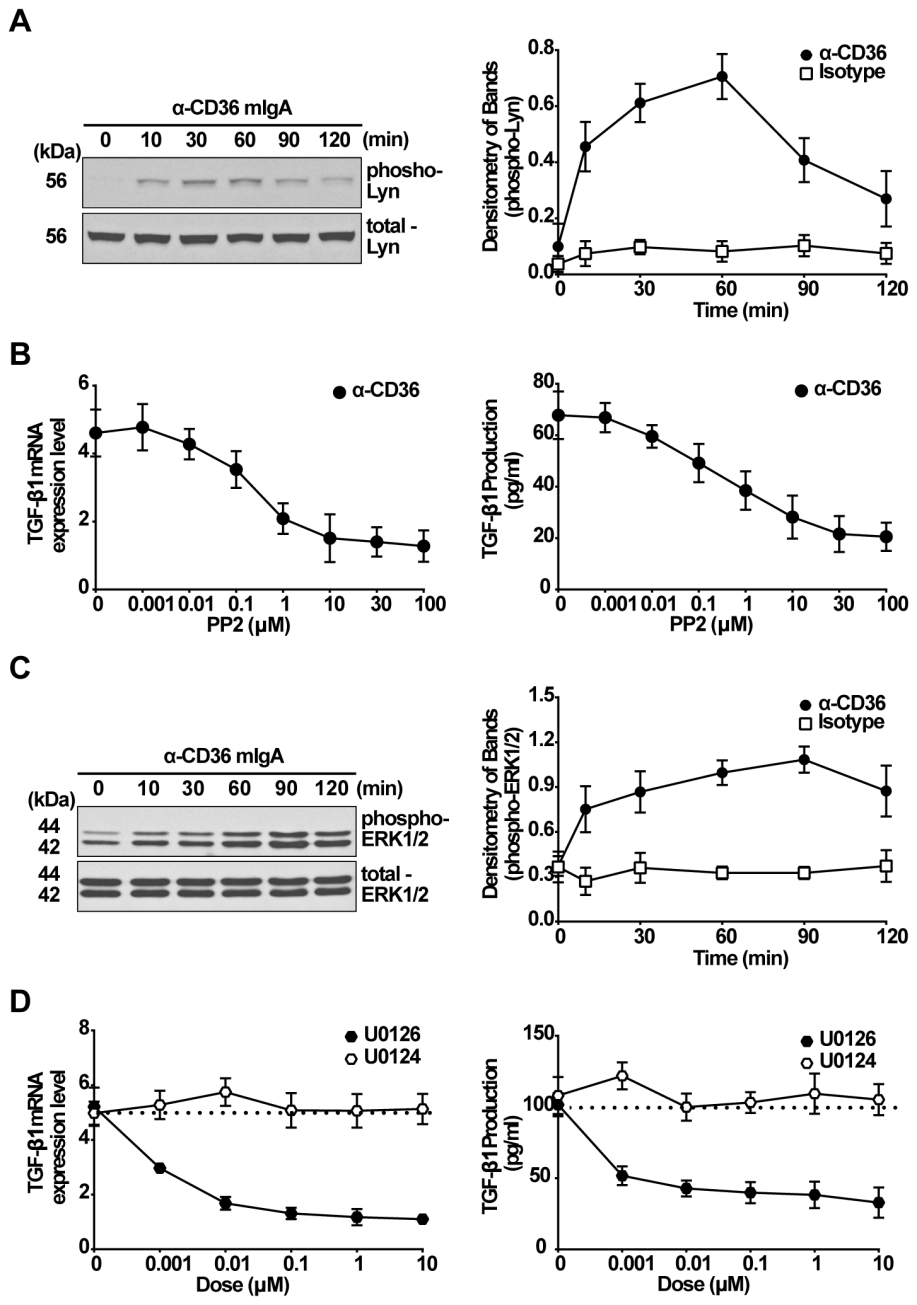
Both Lyn kinase and ERK1/2 MAPK are required for TGF-β1 synthesis induced by activating anti-CD36 mIgA. A, RAWTβRII cells were stimulated with activating anti-CD36 mIgA (2 µg/ml) for the times indicated. Total cell lysates were immunoblotted for phospho-Lyn kinase and the band density was normalized to total Lyn kinase. B, RAWTβRII cells were pretreated with the src-family kinase inhibitor PP2 (0.001 to 100 µM) for 2 h and then stimulated with anti-CD36 mIgA (2 µg/ml). After 6 h, TGF-β1 mRNA expression was analyzed by real-time PCR and normalized to GAPDH. Total TGF-β1 in the conditioned medium was analyzed by ELISA after 18 h. C, A time course of ERK1/2 phosphorylation was assessed by Western blotting in RAWTβRII cells treated with anti-CD36 mIgA (2 µg/ml). Phospho-ERK1/2 band density was normalized to total ERK1/2. D, RAWTβRII cells were preincubated with MEK kinase inhibitor U0126 or inactive analogue U0124 for 2 h and then stimulated with anti-CD36 mIgA for 6 h to detect TGF-β1 mRNA expression or for 18 h to detect secreted TGF-β1 protein as in Figure 1. Values represent means ± SD of six separate experiments.

Lyn kinase has been implicated to be a key effector mediating CD36 signaling [45]. To test its potential role in CD36-mediated TGF-β1 production, we first performed a kinase activation profile. Western blot analysis showed that phosphorylation of Lyn was induced by activating anti-CD36 mIgA in a time response (Figure 4A). To address its potential contribution in mediating CD36 induction of TGF-β1 synthesis, RAWTβRII cells were pretreated with PP2 (2 h with 0.001-100 µM), which has been shown to block Lyn kinase phosphorylation and activity [46,47], prior to addition of activating CD36 mIgA. PP2 significantly inhibited the induced mRNA synthesis and TGF-β1 protein production (Figure 4B). Our previous studies showed that TGF-β inhibits the LPS-induced TNFα production by macrophages *in vitro* [8,18]. Therefore, to demonstrate that the observed differences in TGF-β1 production in the presence of PP2 were physiologically relevant, we measured suppression of TNFα mRNA expression in RAW cells stimulated with LPS in the absence or presence of 20-60 pg/ml TGF-β1. As shown in Figure S2, pretreatment with 60 pg/ml TGF-β1 significantly suppressed TNFα mRNA expression compared to the 20 pg/ml dose, which did not suppress LPS-induced TNFα mRNA expression.

Previous studies had implicated MAPKs in the stimulation of TGF-β generation by apoptotic cells [15]. Accordingly, we next investigated their role in the effects of CD36 stimulation. In RAWTβRII cells, phosphorylation of ERK1/2 was induced by activating anti-CD36 mIgA and shown to be increased with time (Figure 4C). However, phosphorylation of p38, JNK1/2 and Akt were not altered by the activating anti-CD36 antibody (data not shown). To confirm the requirement of ERK1/2, the cells were pretreated with the MEK inhibitor (U0126) or inhibitor analogue (U0124), for 2 h at doses from 0.001–10 µM before stimulation with activating anti-CD36 mIgA. As shown in Figure 4D, TGF-β1 mRNA and secreted protein in the medium were significantly reduced by the MEK inhibitor. By contrast, inhibitors of p38 MAPK, JNK or phosphatidylinositide 3-kinase (PI3K)/Akt showed no effect on TGF-β1 production (data not shown).

**Figure 5:**
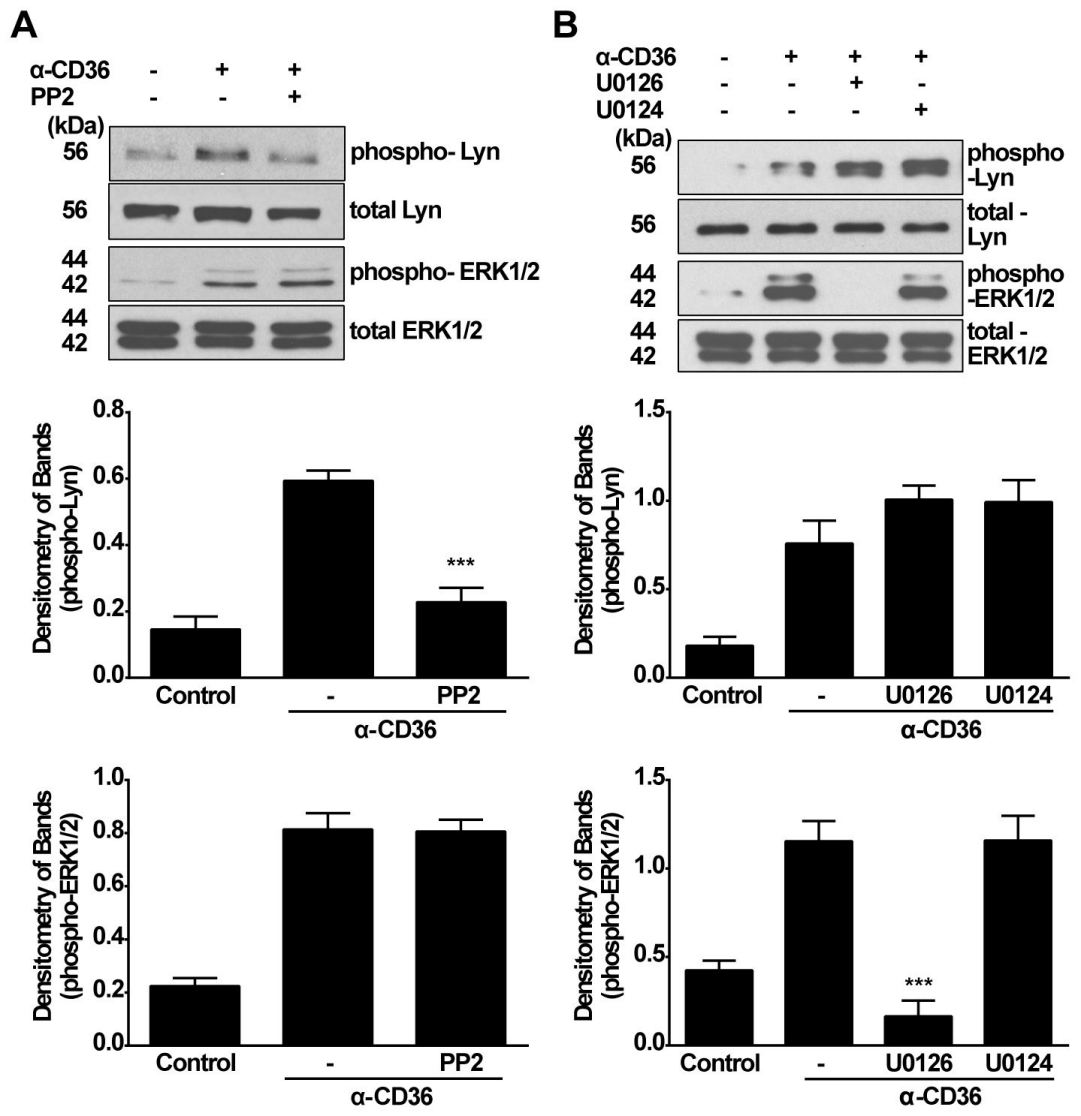
Lyn kinase and ERK1/2 MAPK act by separate pathways for activating anti-CD36 mIgA-induced TGF-β1 synthesis. A, RAWTβRII cells were pretreated with inhibitor PP2 (30 µM) for 2 h prior to stimulation with anti-CD36 mIgA (2 µg/ml) for 60 min. Phosphorylation of Lyn and ERK1/2 were analyzed by Western blotting using total cell lysates. B, RAWTβRII cells were pretreated with inhibitor U0126 or analogue U0124 (1.0 µM) for 2 h and then incubated with anti-CD36 mIgA (2 µg/ml) for 90 min. Total cell lysates were used to analyze phosphorylation of Lyn and ERK1/2. Relative values for phosphorylated kinase versus total kinase were determined by densitometry and expressed as means ± SD of five separate experiments. *** *P*<0.001.

To test the possibility that Lyn and ERK1/2 might act in the same pathway, RAWTβRII cells were pretreated with either PP2 or U0126 prior to incubation with activating anti-CD36 mIgA. Phosphorylation of Lyn and ERK1/2 were detected by Western blot in the treated cells. Surprisingly, as shown in Figure 5A, PP2 blocked the phosphorylation of Lyn but had no effect on ERK1/2 phosphorylation whereas U0126 inhibited ERK1/2 phosphorylation but did not alter Lyn phosphorylation (Figure 5B). This suggested that Lyn and ERK1/2 function independently in the induction of TGF-β1 production.

### TGF-β1 production in response to apoptotic cells was mediated by CD36 signaling via Lyn and ERK1/2

**Figure 6:**
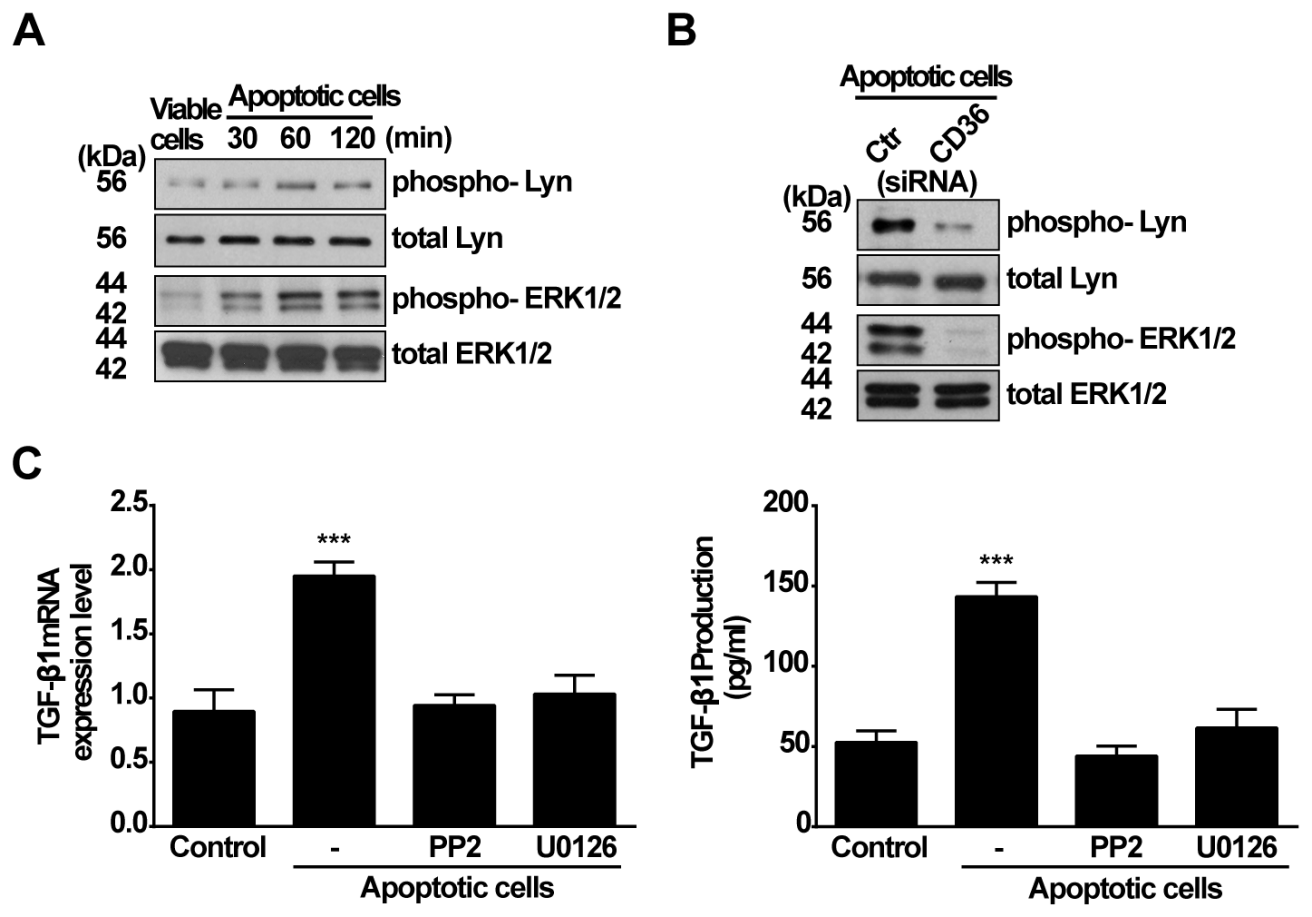
Lyn kinase and ERK1/2 are involved in CD36 mediated TGF-β1 induction by apoptotic cells. A, RAWTβRII cells were cultured in the presence of viable or apoptotic Jurkat cells for the time indicated. Total cell lysates were used for analyzing phosphorylation of ERK1/2 MAPK and Lyn kinase by Western blotting. B, RAWTβRII cells transfected with CD36-target siRNA or control siRNA (Ctr siRNA) for 24 h were incubated with apoptotic Jurkat cells for 60 or 90 min to analyze phosphorylation of Lyn kinase or ERK1/2, respectively. C, RAWTβRII cells were preincubated with PP2 (30 µM) or U0126 (1 µM) for 2h and then co-cultured with apoptotic Jurkat cells. TGF-β1 mRNA expression or secreted TGF-β1 protein was analyzed as in Figure 1. Values represent means ± SD of six separate experiments. ***, *P*<0.001.

As described above, ERK1/2 and Lyn are important signaling pathways, leading to up-regulation of TGF-β1 production (Figure 4). Thus, we examined the involvement of ERK1/2 and Lyn in response to apoptotic cells treatment. As shown in Figure 6A, exposure to apoptotic Jurkat cells resulted in increased phosphorylation of ERK1/2 and Lyn in RAWTβRII cells. As hypothesized, the levels of phosphorylated ERK1/2 and Lyn did not change after exposure to viable cells. Moreover, apoptotic cell-induced phosphorylation of Lyn and ERK1/2 was inhibited by CD36 siRNA treatment (Figure 6B). To further confirm the requirement of Lyn and ERK1/2, RAWTβRII cells were pretreated with PP2 or U0126 for 2 h prior to stimulation with apoptotic cells. As expected, apoptotic cell induced increases in expression of TGF-β1 mRNA and secreted protein were inhibited (Figure 6C). These data suggest that recognition of apoptotic cells, via macrophage CD36, signaled through Lyn and ERK1/2 kinases to up-regulate mRNA expression and production of TGF-β1.

## Discussion

Recognition of phosphatidylserine exposed on the surface of apoptotic cells has been shown to stimulate their uptake and removal, as well as the production of active TGF-β. While numerous receptors and ligands for PS have been implicated in the uptake, those involved in stimulation of TGF-β synthesis have received less attention. Here we have characterized CD36 as an important candidate PS receptor for simulation of TGF-β1 transcription and release of TGF-β1 protein from macrophages.

To explore the mechanisms by which apoptotic cells induce TGF-β1 production, it was first necessary to set up a system to avoid the known auto-induction of TGF-β1 synthesis by TGF-β1 itself using RAW 264.7 cells stably transfected with the dominant negative, truncated TGF-β receptor II [15]. These cells were shown not to respond to added TGF-β1 and, as expected, stimulation with apoptotic cells showed lower overall levels of TGF-β1 produced, thus emphasizing the contribution of such an auto-induction in the overall TGF-β amounts generated when macrophages are stimulated. Nonetheless, as reported previously [7,8,15], apoptotic cells were here shown themselves to be a potent stimulus for the induction of TGF-β1 transcription, translation and secretion by mechanisms dependent on the exposed PS (Figure 1). Examination of the receptor involved in this recognition and stimulation demonstrated a key role for the class B scavenger receptor, CD36. Thus, knockdown of CD36 by siRNA potently prevented the initiation of TGF-β1 transcription and protein production. By contrast, induction of TGF-β1 with other unrelated stimuli such as LPS or PMA was unaffected (Figure 2). CD36 is a multifunctional receptor with known pro- as well as anti-inflammatory properties [33,34,44,45,48,49]. It has also previously been identified as one of many the many, presumably redundant, candidates for the PS-dependent uptake of apoptotic cells [23–26,29,33,34]. However, despite the implication of CD36 effects in inflammatory responses, internalization of apoptotic cells is generally non-inflammatory [33,34] and we here suggest in part that this may be driven by CD36 stimulation of TGF-β1 production.

In support of the ability of CD36 activation to initiate synthesis of TGF-β1, we carried out studies with a stimulatory anti-CD36 antibody (JC63.1). The antibody was a potent stimulus for TGF-β1 production (Figure 3) but interestingly not for another important anti-inflammatory mediator, IL-10 (data not shown). The antibody was not effective on the CD36 deficient cells (Figure 3) and the TGF-β1 mRNA production was not inhibited by polymyxin B ruling out a possible effect of endotoxin contamination (data not shown).

Recent studies, including pharmacologic and genetic knockout experiments, identified signaling pathways downstream of CD36 stimulation that include the non-receptor tyrosine kinases of the Src family [45], especially Lyn [49,50]. Accordingly, examination of Src family kinase phosphorylation showed evidence of Lyn involvement in the activating anti-CD36 induced TGF-β1 production – i.e. induction of Lyn phosphorylation after anti-CD36 antibody stimulation and blockade of TGF-β1 production with the inhibitor PP2 (Figure 4). Although PP2 treatment leads to a small decrease of secreted TGF-β1 production, our TGF-β1 functional assay (Figure S2) indicates that the small changes in TGF-β secretion are physiologically relevant. As previously described [15], stimulation of TGF-β synthesis by apoptotic cells was shown to involve MAPKs. Accordingly, we evaluated MAPK activation (phosphorylation) after stimulation with activating anti-CD36 antibody. Phosphorylation of ERK1/2 increased markedly in RAW cells in response to antibody treatment and peaked at 60 min (Figure 4) and blockade of MEK, the upstream activator of ERKs, with its inhibitor U0126 suppressed apoptotic cell-induced TGF-β1 expression. Activation of MAPKs occurs in the cytoplasm, but to exert many of their actions, they must translocate into the nucleus to interact with transcription factors [51]. MAPKs have cis-acting regulatory elements in the mouse-TGF promoter region, which respond to various transcription factors, including specificity protein-1 and activating protein 1. Thus, it is possible that apoptotic cell-induced TGF-β mRNA expression is mediated through activation of these transcription factors via MAPK signaling. Xiao et al. reported that all of the MAPK members, including p38/ERK/JNK, are required for apoptotic Jurkat cells up-regulation of TGF-β production [15]. In contrast to ERKs, the anti-CD36 antibody treatment did not lead to the phosphorylation of p38 MAPK and JNK1/2 in RAWTβRII cells, and their individual inhibitors did not alter the induced TGF-β1 production (data not shown).

When PS-exposing apoptotic cells were examined, all three MAPKs were activated. However, knock-down of CD36 blocked the activation of ERKs and Lyn (Figure 6), but not JNK1/2 or p38 (data not shown). Again, the production of TGF-β1 in response to the apoptotic cells was susceptible to inhibition of either ERK or Lyn pathway. Activation of a Src family member such as Lyn could lead to subsequent activation of MAP kinases [45]. However, when we examined the relationship between Lyn and ERK1/2 it appeared that they function in separate pathways, both involved in TGF-β1 synthesis (Figure 5).

The present study has begun to address the PS-recognizing receptor and signal pathways involved in enhanced TGF-β synthesis in response to PS-exposing apoptotic cells and implicates a significant role for CD36 in this process. However, the study does not exclude the possible participation of other “PS” receptors in stimulating TGF-β production in other macrophages and/or cell types. Therefore, future studies will be warranted to address whether TGF-β1 production is selectively dependent on receptor CD36 by using primary macrophages (i.e. peritoneal macrophages) from wild type and CD36 knock out mice. Intriguingly, CD36 thereby gains additional relevance for TGF-β production in that it is not only an important participant in activation of secreted latent TGF-β [35,36,38] but in addition in its transcription and translation. 

## Supporting Information

Figure S1 **Expression of phagocytic receptor and bridge molecule candidates in CD36 knock-down cells.**
Total RNA was isolated from RAWTβRII cells treated with CD36-target siRNA or control siRNA (Ctr siRNA). The mRNA expression of CD36, TIM4, MFG-E8, CD91 and C1q, were analyzed by real-time RT-PCR and normalized to GAPDH. Values represent as means ± SD from five independent experiments. *, *P* < 0.05.
(TIFF)

Figure S2 **TGF-β1 suppressed LPS induced TNFα mRNA expression *in vitro*.**
RAW 264.7 cells were pretreated with TGF-β1 (90 min with 0, 20, 40 and 60 pg/ml) prior to stimulation with LPS (1000 pg/ml) for 6 h. TNFα mRNA expression, which was normalized to GAPDH, was analyzed by real-time PCR and represented as fold change. TGF-β1 inhibitory effect on LPS-induced TNFα mRNA expression was expressed as percentage (%) inhibition = {(TNFα mRNA fold change_control_ − TNFα mRNA fold change_sample_)/TNFα mRNA fold change_control_} × 100. Values represent as means ± SD from five independent experiments. ***, *P* < 0.001.
(TIFF)
