# Supplementary figures and images for: Induction of TGF-β1 Synthesis by Macrophages in Response to Apoptotic Cells Requires Activation of the Scavenger Receptor CD36
Source: PLoS One. 2013 Aug 2;8(8):e72772. doi: 10.1371/journal.pone.0072772 (PMC3732218; doi:10.1371/journal.pone.0072772)

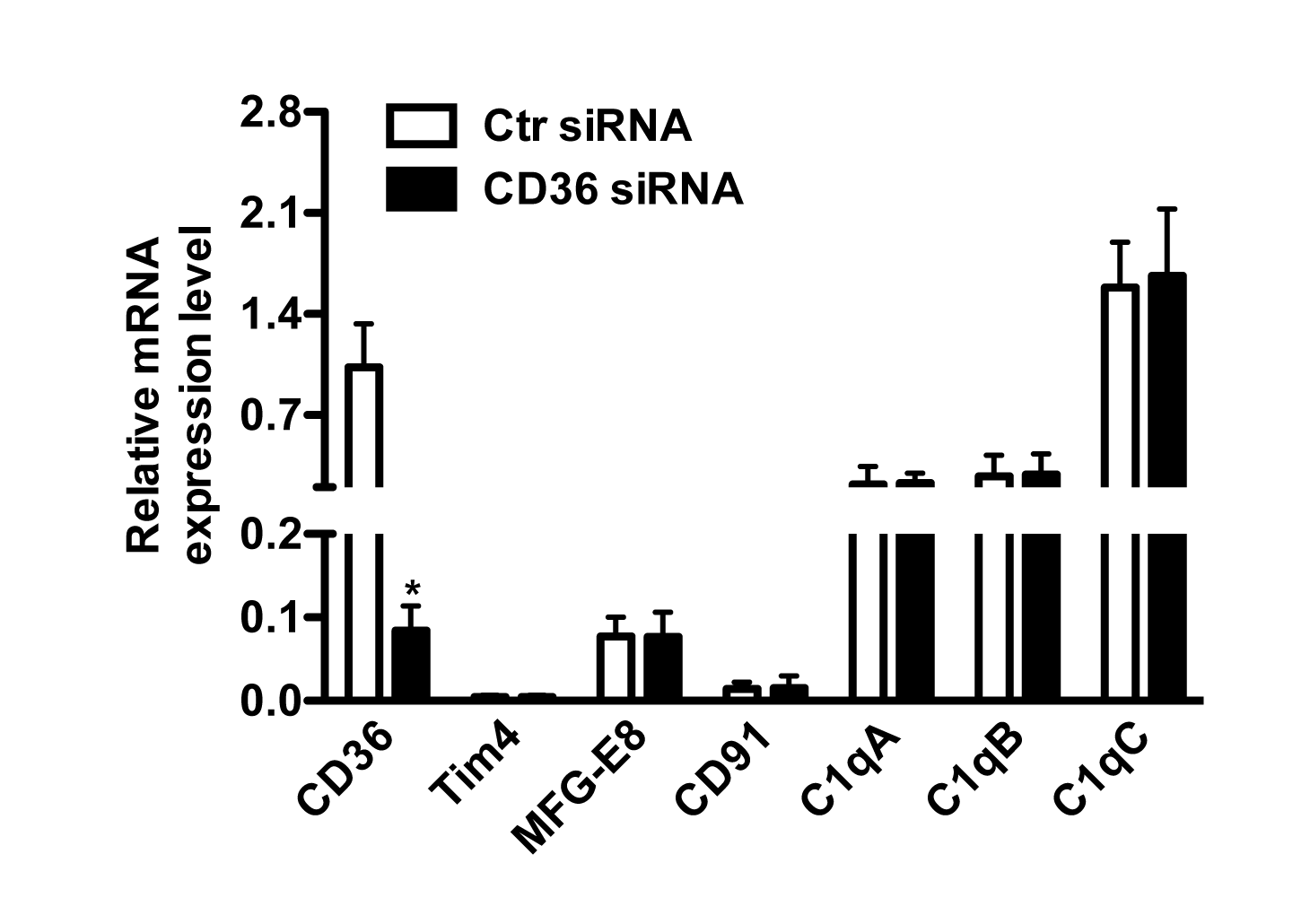

Supplement: Figure S1 — Expression of phagocytic receptor and bridge molecule candidates in CD36 knock-down cells. Total RNA was isolated from RAWTβRII cells treated with CD36-target siRNA or control siRNA (Ctr siRNA). The mRNA expression of CD36, TIM4, MFG-E8, CD91 and C1q, were analyzed by real-time RT-PCR and normalized to GAPDH. Values represent as means ± SD from five independent experiments. *, P < 0.05. (TIFF) [file pone.0072772.s001.tiff]

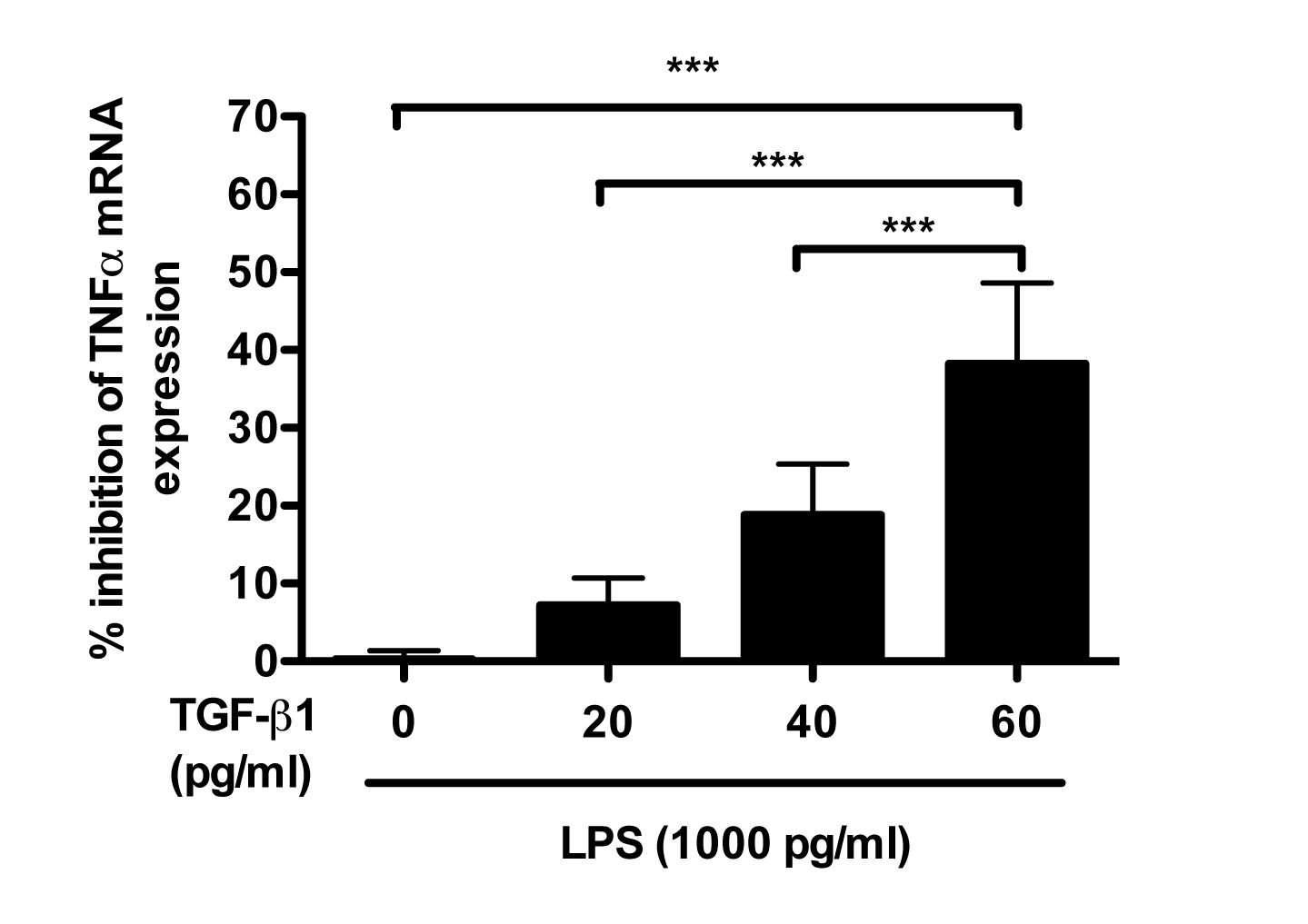

Supplement: Figure S2 — TGF-β1 suppressed LPS induced TNFα mRNA expression in vitro. RAW 264.7 cells were pretreated with TGF-β1 (90 min with 0, 20, 40 and 60 pg/ml) prior to stimulation with LPS (1000 pg/ml) for 6 h. TNFα mRNA expression, which was normalized to GAPDH, was analyzed by real-time PCR and represented as fold change. TGF-β1 inhibitory effect on LPS-induced TNFα mRNA expression was expressed as percentage (%) inhibition = {(TNFα mRNA fold changecontrol − TNFα mRNA fold changesample)/TNFα mRNA fold changecontrol} × 100. Values represent as means ± SD from five independent experiments. ***, P < 0.001. (TIFF) [file pone.0072772.s002.tiff]
